# Reproductive health status of north western Himalayan Gaddi sheep: An abattoir study

**Published:** 2014-11-06

**Authors:** A. Sharma, P. Kumar, M. Singh, N.K. Vasishta

**Affiliations:** *Department of Veterinary Gynaecology and Obstetrics, College of Veterinary and Animal Sciences, Palampur, India*

**Keywords:** Abattoir, Gaddi sheep, Genital abnormalities, Reproductive health status

## Abstract

The study was aimed to provide basic information regarding reproductive status of Gaddi sheep reared by nomadic tribe of Himachal Pradesh. Female genitalia of Gaddi sheep (n=190) were collected from unorganized abattoirs around Palampur over a period of one and half years. Out of total genitalia examined, 80.53% were grossly normal and 19.47% had one or more genital abnormalities. Genital abnormalities were categorized as ovarian (5.26%), uterine (10.53%) and miscellaneous (3.68%). Amongst ovarian abnormalities are follicular cysts (3.16%) and ovaro-bursal adhesions (2.10%), which were recorded in Gaddi ewes. Uterine abnormalities include hydrometra (4.74%), pyometra (2.63%), mucometra (2.10%), endometritis (0.53%) and mummification (0.53%). Miscellaneous abnormalities include parovarian cysts (2.10%), parasitic cysts (1.05%) and nodules on both uterine horns (0.53%). Among the genital abnormalities in sheep, highest incidence (24.32%) was observed with hydrometra and lowest (2.7%) with each of endometritis, mummification and nodular growth on both uterine horns. Thus the uterus (54.07%) was most commonly affected, followed by the ovary (27.02%) and miscellaneous (18.91%) in ewes. In present study, 8.95% pregnant sheep were also slaughtered, with fetal age in majority of cases two months or less on the basis of CRL measurement which represents a huge economic loss.

## Introduction

Gaddi sheep is one of the recognized breeds reared mainly in Kangra and Chamba districts of Himachal Pradesh, India. It is usually considered as dual purpose breed, well adapted to migration and is least susceptible to many of the known diseases occurring in exotic or crossbred sheep. The most important factor determining the success of sheep production is its reproductive efficiency which in turn is adversely affected by different reproductive diseases. Although various methods can be used to detect genital tract abnormalities viz. ultrasonography, radiology, laboratory diagnosis and ante mortem or post mortem examination of abattoir animals, the abattoir provides a useful source of information on the types and prevalence of such abnormalities and allows for examination of a large number of animals in a short time at a very low cost (Palmeiri *et al.*, 2011).

Lack of proper ante mortem examination or inadequate facilities and little information available on the extent of such reproductive wastage may be the probable reasons to assess the prenatal losses. Keeping in mind the importance of migratory Gaddi sheep practice, its vast potential as an industry and future prospects for self-employment, the present study was planned to critically evaluate and document the reproductive problems and assess the prenatal losses occurring in Gaddi sheep of Himachal Pradesh.

## Materials and Methods

The Gaddi sheep genitalia were collected from abattoirs around Palampur from September 2010 to March 2012, irrespective of age and reproductive status at the time of slaughter. Whole of the genital tract was collected in normal saline (0.9%) immediately after slaughter. It was washed with normal saline and transported to the laboratory in separate thermocoal containers at 37°C and examined within 2 hours of slaughter. Gross morphological examination of genitalia was carried out. The detailed examination of ovary and oviduct was performed to detect abnormalities and presence or absence of follicle and/or corpus luteum (CL).

Ovaries with follicles ≥ 10 mm diameter were considered cystic (Winter and Dobson, 1992). Based on appearance, the cysts were classified into follicular and luteinised cysts. Subsequently, the genital tracts were incised along the longitudinal axis of cervix, uterine body and uterine horns. Uterus was examined for evidence of accumulation of fluid in one or both horns. Depending upon the colour, consistency and viscosity of the uterine contents viz. watery, mucoid or pus mixed, the condition was classified as hydrometra, mucometra or pyometra, respectively. These genitalia were examined for normal physiological status or abnormality or disease condition and classified as normal, gravid or abnormal. The detailed examination of pregnant uterus was carried out to record the number and location of fetus/es. The age of fetus was estimated using formula: X=2.1(Y+17), where X is the developmental age in days and Y is crown-anus length in centimeters (Arthur *et al.*, 2001).

## Results

The present study was designed to conduct abattoir survey on genital status and to document various reproductive disorders in Gaddi sheep (n=190) of Himachal Pradesh. The normal genital status and the prevalence of various types of reproductive tract disorders of Gaddi sheep are summarized in Table 1.

**Table 1:** Genital status and prevalence of reproductive abnormalities in Gaddi sheep abattoir genitalia (n=190).

Genital Status	Location	Disease Condition	Number (%)
Abnormal (n=37; 19.47%)
	Ovary	Follicular Cysts	6 (3.16)
		Ovarobursal adhesions	4 (2.10)
	Total		10 (5.26)
	Uterus	Endometritis	1 (0.53)
		Pyometra	5 (2.63)
		Hydrometra	9 (4.74)
		Mucometra	4 (2.10)
		Mummification	1 (0.53)
	Total		20 (10.53)
	Miscellaneous	Parasitic Cyst	2 (1.05)
		Par Ovarian Cyst	4 (2.10)
		Nodules on both horns	1 (0.53)
	Total		7 (3.68)
Normal (n=153; 80.53%)	Pregnant		17 (8.95)
	Non pregnant		136 (71.58)

**Fig. 1:**
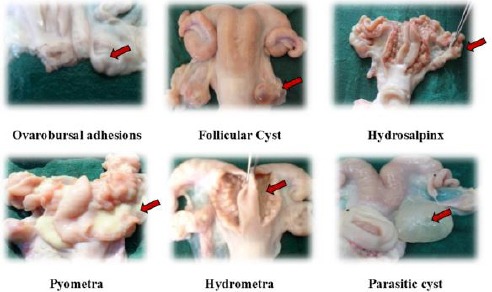
Various genital abnormalities observed in Gaddi sheep abattoir specimens.

**Fig. 2:**
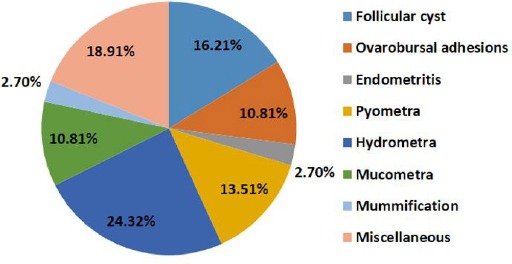
Distribution (%) of reproductive abnormalities of Gaddi sheep (n=37).

Out of total sheep genitalia examined, 80.53% (n=153) were physiologically normal and 19.47% (n=37) had one or more genital abnormalities (Fig. 1). Among ovarian abnormalities (5.26%), follicular cysts were recorded in 3.16%, of which 66.66% were on the right ovary and 33.33% were on left ovary. Ovarobursal adhesions were observed in 2.10%, of which 50% were unilateral (each on either ovary) and 50% were bilateral. Severity of adhesions varied from a few fibrin strands between the ovary and mesosalpinx to complete adhesion involving the entire ovary and part of the surface of urinary bladder. Uterine abnormalities constitute endometritis (0.53%), pyometra (2.63%), mucometra (2.10%), hydrometra (4.74%) and mummification (0.53%). Among the genital abnormalities in sheep, highest incidence of hydrometra (24.32%) and lowest of endometritis, mummification and nodular growth on both uterine horns (2.7% each) were recorded. Thus the uterus was most commonly affected, accounting for 54.05% of the abnormalities, followed by the ovary 27.02% in ewes (Fig. 2).

Various other abnormalities such as parovarian cysts (2.10%) were also detected, and were generally single but double and triple cysts were also recorded. Cysts were ranging from 3 mm to 8 mm in diameter and most of them were located on the outer surface of uterine horns. The cysts were thin-walled, tense and contained clear watery fluid. Two cases (1.05%) of parasitic cyst (*Cysticercus tenuicolis*; Fig. 1) and one case (0.53%) of nodules on horns were also detected in ewes.

In present study, pregnancy was observed in 8.95% of genital tracts and most of the fetuses (n=13; 76.47%) were estimated to be less than two months old (ranging between 38 to 54 days) and only one fetus (5.88%) had gestational age of 72 days. Out of the 17 gravid uteri, 23.52% (n=4) had early pregnancy. Single pregnancy (94.11%) predominates bilateral pregnancy (5.88%) in our study. The incidence of left horn and right horn pregnancy was 47.06% each. Transuterine migration was observed in two cases (11.76%) evidenced by presence of CL on ovary contralateral to presence of fetus. When applied on a nationwide basis, this rate of slaughter of pregnant sheep and population represents a considerable loss in terms of production and income.

## Discussion

Higher incidence of gross genital abnormalities in ewes to those recorded in present study were earlier reported in the UK (23.2%; Winter and Dobson, 1992), Iran (25.8%; Moghaddam and Gooraninejad, 2007), Ethiopia (22.2%; Regassa *et al.*, 2009) and Iraq (25.7%; Dawood, 2010). A lower incidence of 3.3%; (Smith *et al.*, 1998), 6.52% (Sudhakar *et al.*, 2010) and 16.6% (Khodakaram and Davari, 2013) have also been recorded and the differences may be due to sample size, breed and the environmental variations in different places.

Ovarian abnormalities were observed in 5.26% of ewes in the present study. But simultaneously higher incidence (20.1%) of ovarian abnormalities in ewes have earlier been recorded by Moghaddam and Gooraninejad (2007). Smith *et al*. (1999) observed that ovarian abnormalities were 3.36% of the total acquired abnormalities in the ewes and 10.68% of those in the nulliparous animals. Follicular cysts were recorded in 3.16% in the present study. Lower incidence (0.3%, 0.12%, 1.85% and 0.81%) of follicular cysts in ewes has been reported earlier by Alosta *et al*. (1998), Moghaddam and Gooraninejad (2007), Sudhakar *et al*. *(*2010) and Khodakaram and Davari (2013), respectively.

However, higher incidence (4.3%) of ovarian cysts in Ethiopian highland ewes has also been recorded (Regassa *et al.*, 2009). Although the significance of ovarian cysts in the ewe is not very well known, yet it has been a common finding in different breeds of ewes. Similar incidence of ovarobursal adhesions has been reported earlier as 2.8%; (Dawood, 2010) and 2.3%; (Alosta *et al.*, 1998). Moghaddam and Gooraninejad (2007) recorded a higher incidence (11.5%) of adhesions in Awassi ewes. Whereas lower incidence of 0.14, 1.2 and 0.49% have also been reported by Emady *et al*. (1975); Long (1980) and Sudhakar *et al*. (2010), respectively.

Uterine abnormalities recorded in present study include endometritis (0.53%), pyometra (2.63%), mucometra (2.10%), hydrometra (4.74%) and mummification (0.53%). Similar incidence (0.49%) of endometritis in ewes has earlier been documented (Sudhakar *et al.*, 2010). However, higher incidence of endometritis viz. 2.3% (Alosta *et al.*, 1998), 2.93% (Khodakaram and Davari, 2013) and 24.8% (Dawood, 2010) has also been reported and the variation in the prevalence of endometritis may probably be due to differences in the management system under which the animals were maintained. Slightly lower incidence has earlier been recorded as 0.2% (Khodakaram and Davari, 2013), 0.7% (Winter and Dobson, 1992) and 1.9% (Dawood, 2010).

Pyometra may arise as a sequel to chronic endometritis (Arthur *et al.*, 1989) or as a result of infection subsequent to mucometra (Winter and Dobson, 1992). Winter and Dobson (1992) reported mucometra in 3.76% of the genital tract of culled ewes. Lower incidence of 0.2% was also observed by Moghaddam and Gooraninejad (2007). Mucometra was commonly recognised as a consequence of the chronic increase in the concentration of circulating steroids that occurred in cases of cystic ovarian diseases (Roberts, 1971). Comparable incidence (4.13%) of hydrometra in ewes was observed by Winter and Dobson (1992), whereas a lower incidence of hydrometra at 0.15% (Khodakaram and Davari, 2013) and 0.3% (Moghaddam and Gooraninejad, 2007) was also reported in Awassi ewes. A low incidence of 0.03% of mummified fetus (Smith *et al.*, 1999) in culled ewes was reported.

Mummified fetuses may be macerated, expelled spontaneously before or after term or even retained indefinitely (Acland, 2001). No specific cause of mummification could be established in the present study. Moghaddam and Gooraninejad (2007) observed that the highest rate of abnormalities was in the ovaries, followed by ones in the oviduct and the uteri of abattoir Awassi ewes genitalia examined. Lower incidence of parovarian cysts (0.9%) and *Cysticercustenuicolis* cysts (0.15%) have earlier been reported by Khodakaram and Davari (2013).

Similar incidence of pregnancy (10% and 12.3%) was also reported by Alosta *et al*. (1998) and Khodakaram and Davari (2013), but comparatively, higher incidence (21.4% and 55.2%) of pregnancy in slaughtered ewes was recorded by Moghaddam and Gooraninejad (2007) and Regassa *et al*. (2007), respectively. Contrarily, lower incidence (3.37%) in cull ewes has been documented (Smith *et al.*, 1999). Presumably, the migration in polytocous animals equalises the distribution of embryos within the uterus despite that the ovaries contribute unequally to total number of embryos (Scanlon, 1972; Reimers *et al.*, 1973) and hence is of immense significance in minimizing early embryonic losses.

Approximately similar incidence (12.8% and 11.5%) of transuterine migration in sheep has been documented earlier (Emady, 1976; Wilson and Traore, 1988), respectively. Contrarily, Emady (1976) reported that out of 74 pregnant ovine uteri, there were 34 migrations from left horn to right and 40 from right horn to left horn. A lower incidence (8.9%) of transuterine migration (4.9%; right to left and 4%; left to right) in ewes has also been recorded (Moghaddam and Gooraninejad, 2007). Alosta *et al*. (1998) recorded that embryonic migration was observed in 12% of the single ovulating ewes (66.7%; right to left and 33.3%; left to right) and in all ewes pregnant with twins had double ovulations on one ovary.

In conclusion, the uterus was most commonly affected (54.07% abnormalities), followed by the ovary (27.02%) and miscellaneous (18.91%) in ewes. Among the genital abnormalities in Gaddi sheep, highest incidence of hydrometra (24.32%) and lowest of endometritis, mummification and nodular growth on both uterine horns (2.7% each) were recorded. Pregnant ewe (8.95%) of approximately two months gestations were also slaughtered along with other animals due to lack of ante mortem pregnancy diagnosis performed at unorganised slaughter houses. When applied on a nationwide basis, this rate of slaughter of pregnant sheep and population represents a considerable loss in terms of production and income.
